# Chronic Posttraumatic Stress Disorder and Comorbid Cognitive and Physical Impairments in World Trade Center Responders

**DOI:** 10.1002/jts.22631

**Published:** 2020-11-21

**Authors:** Erica D. Diminich, Sean A. P. Clouston, Alexandra Kranidis, Minos Kritikos, Roman Kotov, Peifen Kuan, Melissa Carr, Evelyn J. Bromet, Benjamin J. Luft

**Affiliations:** ^1^ Program in Public Health Renaissance School of Medicine at Stony Brook University Stony Brook New York USA; ^2^ American Lung Association Hauppauge NY USA; ^3^ Department of Psychiatry Renaissance School of Medicine at Stony Brook University Stony Brook New York USA; ^4^ Department of Applied Mathematics and Statistics Stony Brook University Stony Brook New York USA; ^5^ World Trade Center Responder Health and Wellness Program Department of Medicine Renaissance School of Medicine at Stony Brook University Stony Brook NY USA

## Abstract

Posttraumatic stress disorder (PTSD) has been linked to increased prevalence and incidence of cognitive and physical impairment. When comorbid, these conditions may be associated with poor long‐term outcomes. We examined associations between chronic PTSD and symptom domains with cognitive and physical functioning in World Trade Center (WTC) responders nearly 20 years after the September 11, 2001, terrorist attacks. Participants included a cross‐sectional sample of 4,815 responders who attended a monitoring program in 2015–2018. Montreal Cognitive Assessment scores less than 23 indicated cognitive impairment (CogI); Short Physical Performance Battery scores 9 or lower on a hand‐grip test indicated physical impairment (PhysI). Comorbid cognitive/physical impairment (Cog/PhysI) was defined as having cognitive impairment with at least one objective PhysI indicator. Clinical chart review provided PTSD diagnoses; symptom domains were assessed using the PTSD Checklist. Participants were on average 53.05 years (*SD* = 8.01); 13.44% had PTSD, 7.8% had CogI, 24.8% had PhysI, and 5.92% had comorbid Cog/PhysI. Multivariable‐adjusted multinomial logistic regression demonstrated that Responders with PTSD have more than three times the risk of Cog/PhysI (adjusted *RR* = 3.29, 95% CI 2.44‐ 4.44). Domain‐specific analyses revealed that emotional numbing symptoms predicted an increased risk of PhysI (adjusted *RR* = 1.57, 95% CI 1.08‐2.28), whereas reexperiencing symptoms were associated with comorbid Cog/PhysI (adjusted *RR* = 3.96, 95% CI, 2.33‐6.74). These results suggest that responders with chronic PTSD may have increased risk of deficits beyond age‐expected impairment characterized by the emergence of comorbid Cog/PhysI at midlife.

Nearly two decades after the September 11, 2001, terrorist attacks on the World Trade Center (WTC) in New York City (9/11), first responders and nontraditional rescue and recovery workers and volunteers, hereafter referred to as “WTC responders” or “responders,” continue to bear a high burden of persistent WTC‐related psychiatric symptoms, including posttraumatic stress disorder (PTSD). In the aftermath of the attacks, WTC responders experienced severe psychological stressors while engaged in rescue and recovery operations, including witnessing people jumping from the burning towers and digging through the rubble for survivors and victims’ belongings (Dasaro et al., 2017). Many responders endured long hours on‐site, inhaling burning jet fuel, chemicals, and pulverized dust from the collapsed towers (Stellman et al., 2008). Responders are currently in their mid‐fifties on average, and many are now experiencing cognitive dysfunction common in neurodegenerative conditions (Clouston, Diminich, et al., 2019).

Previous research has estimated that as many as one‐fifth of WTC responders met the screening criteria for PTSD in the aftermath of exposure and nearly half developed WTC‐related chronic stress symptoms, which persisted for years (Bromet et al., 2016). Posttraumatic stress disorder is heterogeneous in presentation and comprises distinct symptom clusters, including those related to avoidance, hyperarousal, and reexperiencing of trauma‐related reminders, thoughts, and feelings (American Psychiatric Association [APA], 2013). Recent studies have further highlighted the complexity of the disorder and challenges in its treatment, demonstrating regional differences in the brain (Fenster et al., 2018), advanced epigenetic aging (Miller et al., 2018), and increased functional impairment (Shea et al., 2010) associated with distinct symptom clusters.

Individuals with PTSD commonly experience a number of comorbid and debilitating medical conditions and psychiatric disorders, including cardiovascular disease (Beristianos et al., 2016) and major depressive disorder (Flory & Yehuda, 2015). Further, PTSD has been associated with increased impairment across a number of domains, including cognition and physical mobility, which drastically interfere with quality of life (Schurr et al., 2006). Collectively, research conducted predominantly in samples of older adults and veteran populations has indicated that PTSD is independently associated with cognitive deficits (Schuitevoerder et al., 2013) and a higher incidence of self‐reported physical disabilities (Lippa et al., 2015; Samuelson et al., 2017; Wrocklage et al., 2016). However, studies to date have not examined the associations between chronic posttraumatic stress and both cognitive functioning and objective assessments of physical mobility in adults during midlife, when functioning should be relatively intact. The average age of responders during the WTC search, recovery, and rehabilitation operations was 39 years. Developing a nuanced understanding of the impact that WTC‐related PTSD has on long‐term cognitive and physical health is critical to inform treatment, reduce the burden of disease, and enhance quality of life in aging responders.

Current neurobiological models of PTSD indicate that hypothalamic–pituitary–adrenal (HPA) axis hyperactivity and persistent stress symptoms result in a gradual degradation of the brain and body (de Quervain et al., 2017; Stuller et al., 2012). Increasingly, PTSD has been linked to inflammation (Miller et al., 2018) as well as cardiovascular, metabolic, and systemic changes, which, in turn, may accelerate aging processes and result in premature aging‐related conditions, such as dementia (Flatt et al., 2018) and frailty. In line with the results of neuroimaging (Greenberg et al., 2014) and epidemiological studies (Dohrenwend et al., 2006; Marmar et al., 2015) of older veterans and combat‐exposed individuals that have demonstrated that chronic stress is related to poor cognitive performance and functional impairment, accumulating evidence from our research group indicates that WTC responders, who have an average age of 53 years at present, are experiencing a higher incidence of disease and disability compared to older, unexposed adults from the general population (Clouston et al., 2019) which may reflect early senescence. For example, telomere length, a marker of cellular aging associated with the severity of posttraumatic stress symptoms (Shalev et al., 2014) and early mortality in older adults (Epel et al., 2009) was found to be longitudinally associated with declines in pulmonary functioning (Clouston, Edelman, et al., 2019). Responders with PTSD have been shown to exhibit poorer performance on cognitive assessments (Clouston et al., 2016) and increased deficiencies in motor tasks indicative of aging‐related processes, such as chair rise and grip strength (Clouston, Guralnik et al., 2017; Mukherjee et al., 2019), than those without PTSD.

Emerging work across animal models and human neuroimaging studies that examines the mechanisms between PTSD and cognitive and physical impairments demonstrates that unremitting psychological stress contributes to structural and functional brain changes via inflammatory and cellular processes (Shalev et al., 2014) as well as reduced peripheral β‐amyloid distribution (Clouston, Deri, et al., 2019) and increased β‐amyloid deposition and hyperphosphorylated tau burden in the brain in animal models (Baglietto‐Vargas et al., 2015; Devi et al., 2010; Rissman et al., 2007; Rothman et al., 2012) and in vivo studies in humans (Mohamed et al., 2018, 2019; Morgese et al., 2017). Increased deposition of β‐amyloid and protein‐tau are well‐established pathological features of Alzheimer's disease (Jack et al., 2018). Thus, PTSD has increasingly been examined as a risk factor for dementia (Qureshi et al., 2010; Yaffe et al., 2010).

Although researchers have long recognized that cognitive and physical impairments frequently co‐occur, these associations have primarily been studied in older populations (Boyle et al., 2009, 2010) that have utilized self‐reported assessments of physical functioning. The risks of both physical and cognitive disabilities tend to increase with age (Nahhas et al., 2010; Singh‐Manoux et al., 2012) and their co‐occurrence is generally indicative of poorer health outcomes (Yu et al., 2019). However, little is known about how deficits might be exacerbated when examined in concert with posttraumatic stress symptoms.

One interpretation of the association between PTSD and earlier onset of both cognitive and physical impairments (CogPhysI) among WTC responders may be that chronic posttraumatic stress exacerbates the normal aging process, resulting in an age‐related disease characterized by independent increases in the risk of both cognitive impairment (CogI) and physical impairment (PhysI). Conversely, if PTSD causes a condition characterized by comorbid Cog/PhysI, PTSD could be expected to predict an increased risk of comorbid Cog/PhysI in particular. To address this question, we examined the associations between chronic PTSD and the risk of CogI, PhysI, and comorbid Cog/PhysI among a well‐characterized cohort of highly exposed WTC responders at midlife. First, we sought to identify differences in functioning (i.e., unimpaired, CogI, PhysI, comorbid Cog/PhysI) between WTC responders across sociodemographic factors and motor tasks (i.e., walking speed, balance, chair‐rise speed, hand‐grip strength) associated with aging and cognition. We then examined the prevalence of limitations across these domains among responders with comorbid Cog/PhysI. Next, we explored how PTSD and symptom domains (i.e., reexperiencing, avoidance, hyperarousal, and emotional numbing) might be associated with different risks of (a) PhysI without CogI, (b) CogI without PhysI, and (c) comorbid Cog/PhysI.

## Method

### Participants and Procedure

The 9/11 attacks at the WTC in New York City exposed over 100,000 individuals working at Ground Zero, the former site of the WTC, to a range of environmental toxins, including the inhalation of dust, fumes, and airborne particulate matter. In addition to hazardous working conditions, WTC responders were engaged in a number of highly distressing and traumatizing tasks, such as sifting through debris for human remains and transporting bodies and victims’ belongings for identification. Thousands of responders were on‐site within minutes of the attacks and most remained at the WTC site through June 2002, when clean‐up efforts were complete. As a result, and given the unknown long‐term effects that WTC‐related exposures might have on responders and survivors, in 2002, the U.S. Centers for Disease Control and Prevention (CDC) established the WTC Health Program (WTC‐HP), a federally funded program involving eight clinics throughout New York, to provide annual monitoring and treatment for WTC‐related conditions.

The WTC‐HP at Stony Brook University is an open‐enrollment program. Enrollees complete visits every 12–18 months. In 2014, the program at Stony Brook University incorporated cognitive screenings, followed in 2015 by assessments including objective indicators of physical functioning to monitor aging‐related changes among enrollees. In total, 4,827 WTC responders completed both cognitive and physical functional assessments. Due to small numbers, responders with a history of stroke (*n* = 6) were excluded from these analyses, and individuals who lacked information from other covariates were also excluded (*n* = 6).

Participants in the current investigation represented 72.3% of the responders who attended monitoring visits. The final sample was composed of 4,815 responders, most of whom were male (91.1%) and non‐Hispanic White (71.4%). Over one‐quarter of the participants (26.2%) had a college degree. The mean participant age was 51 years at the time of assessment. More than half of the sample (55.5%) reported having spent more than 5 weeks on the debris pit/pile while on‐site at the WTC, and most individuals reported a low exposure severity while on‐site at the WTC. The institutional review board at Stony Brook University approved all procedures. All participants provided informed consent.

### Measures

#### Demographic Characteristics

Demographic characteristics were collected, including age in years, sex (female vs. male); race/ethnicity, categorized as non‐Hispanic White, non‐Hispanic Black, non‐Hispanic other, and Hispanic; educational attainment, categorized as high school or less, some college, and university degree; and information on diagnoses of diabetes, hypertension, and heart problems.

#### Cognitive Impairment

Cognitive impairment was measured using the Montreal Cognitive Assessment (MoCA; Nasreddine et al., 2005), a brief clinician‐administered assessment. The MoCA, which was administered by trained clinical research staff, is an objective and sensitive measure of cognitive function, with high sensitivity and specificity in detecting CogI and dementia (Freitas et al., 2013; Ismail et al., 2010; Luis et al., 2009). The assessment consists of a brief 30‐point screening of eight cognitive domains that can be completed in 10–15 min. A cutoff score of less than 23 was used to indicate CogI (Clouston, Diminich, et al., 2019). Prior work has found a much higher sensitivity and specificity (96% and 95%, respectively) when using a cutoff score of 23 compared to a score of 26 (Luis et al., 2009).

#### Physical Functioning

In 2015, trained clinical research staff administered four brief, validated assessments of physical functioning and impairment, three of which are components of the Short Physical Performance Battery (SPPB; Guralnik et al., 1994). Assessments were categorized using validated cut points to define PhysI, namely (a) walking speed (< 0.8 m/s; Lauretani et al., 2003), (b), chair‐rise speed (>13 seconds; Whitney et al., 2005), and (c) the inability to hold three balancing positions for a minimum of 10 s. Performance assessments were scored according to the standard scoring algorithm for the SPPB, with scores ranging from 0 to 12. Scores of 12 indicate good functioning (Guralnik et al., 2000), and scores of 9 or lower have good sensitivity and specificity in relation to detecting frailty in older adults (da Câmara et al., 2013) and were used to operationalize functional impairment in the present study. We further assessed reductions in hand‐grip strength (kg), a validated biomarker of aging (Sanderson & Scherbov, 2014), by measuring maximal grip across two trials of grip strength in both hands using a Vernier (Beaverton, OR) digital hand grip dynamometer. Maximal grip strength across all trials was reported. Responders were categorized as having low grip strength if their maximal grip strength fell 1.5 standard deviations below the sex‐specific mean grip strength (i.e., 57.4 lb for men, 36.1 lb for women) reported in this population (Mukherjee et al., 2019).

#### WTC‐related Posttraumatic Stress

The 17‐item PTSD Checklist–Civilian Version (PCL; Blanchard et al., 1996), administered at enrollment and each monitoring visit, was used to identify WTC responders with probable WTC‐related PTSD. To reduce the potential for PTSD symptoms to be subject to reverse causation, PCL scores from the assessment most proximal to the enrollment wave were used in the present analyses. Each symptom is rated using a 5‐point scale ranging from 1 (*not at all*) to 5 (*extremely*). Items were summed, with a total score range of 17 to 85. Scores of 44 or higher were used to indicate WTC‐related probable PTSD. The PCL is a validated measure based on the PTSD criteria given in the fourth edition of the *Diagnostic and Statistical Manual of Mental Disorders* (*DSM*‐*IV*) and is used to assess four underlying symptom domains, including reexperiencing, avoidance, emotional numbing, and hyperarousal symptoms (King et al., 1998). The internal reliability of the PCL in this sample was excellent (Cronbach's α = .95), and the measure has been shown to be very good at identifying clinically diagnosed PTSD (area under the curve [AUC] = .86; Blanchard et al., 1996). In the present sample, Cronbach's alpha values for the PTSD symptom domains of reexperiencing, avoidance, emotional numbing, and hyperarousal were .96, .94, .97, and .98, respectively. For the present analyses, PCL scores from participants’ first monitoring visit, which occurred an average of 9.09 years before the cognitive and physical assessments, were utilized.

#### World Trade Center Exposures

Prior work has identified increased risks of pulmonary conditions (Wisnivesky et al., 2011) and cancer (Li et al., 2012) in relation to known exposures to the WTC site dust cloud, as measured using a validated self‐report measure of exposure history (Liu et al., 2014). To determine the pulmonary and carcinogenic exposure severity for participants in the present study, we replicated the continuous exposure severity metric identified by Li et al. (2012). Because much of this metric is weighted toward dust cloud exposures but exposures that affect neurological functioning are likely to differ in size and specification as compared to pulmonary exposures, we additionally accounted for the length of time spent working on the pit/pile to create a proxy for exposure to fine particulate matter that was most intense in the weeks and months after the first post‐9/11 rainfall (Lioy & Georgopoulos, 2006). Because PTSD may be more likely to result when individuals are injured, we identified whether responders reported, at enrollment, any physical injury at the WTC site that was severe enough to require medical attention. Finally, we examined whether individuals reported having any head injuries at the WTC site, using data from multiple sources, including a structured survey, detailed medical history, and clinical records that identified diagnoses of head trauma.

### Data Analysis

Percentages or mean values and standard deviations were calculated to describe the sample. The prevalence estimates for CogI were calculated by age group for descriptive purposes; 95% confidence intervals were also calculated. Next, we examined the prevalence of reduced functioning across motor tasks (i.e., walking, balance, chair‐rise, hand‐grip strength) that might be uniquely associated with the severity of co‐occurring Cog/PhysI. Because the outcome is nominal in nature, multinomial logistic regression (Long & Freese, 2006) was used to identify associations with and predictors of PTSD with and (a) PhysI without CogI, (b) CogI without PhysI, and (c) co‐occurring Cog/PhysI. Multivariable‐adjusted relative risks (a*RR*) are reported alongside 95% confidence intervals and accompanying *p* values. McFadden's adjusted pseudo‐*R* ^2^ was used to indicate model fit. To determine the extent to which comorbid Cog/PhysI was associated with PTSD diagnostic status versus symptom domains, we ran two demographic‐adjusted models examining (a) PTSD diagnosis and (b) PTSD symptom clusters are reported. All model assumptions were tested: No multicollinearity was identified, Cook's distance identified no influential data, and logistic linearity was supported. Previous work has shown that well‐established risk factors for neurodegenerative disorders, including cardiovascular disease, diabetes, and educational attainment, did not explain the associations between PTSD and PhysI (Clouston, Guralnik, et al., 2017) or CogI (Clouston et al., 2016). Because these are well‐established correlates, we included these factors in the fully adjusted models. The analyses were completed using Stata (Version 15).

## Results

Demographic information can be found in Table 1. Of the 4,815 responders included in the present study, 655 (13.60%, 95% CI [12.65%, 14.60%]) had CogI and 1,480 (30.73%, 95% CI [29.44%, 32.06%]) had PhysI. The average participant age was 53.05 years (*SD* = 8.01), and 13.44%, 95% CI [12.49, 14.44] of the sample had PTSD. Table 1 shows the sample characteristics stratified into responders with CogI only (*n* = 375), PhysI only (*n* = 1,200), comorbid Cog/PhysI (*n* = 280), and neither CogI nor PhysI (*n* = 2,960). In total, 5.82%, 95% CI [5.17%, 6.51%], of responders had comorbid Cog/PhysI, which represented 42.75%, 95% CI [38.92%, 46.64%], of those with CogI. The risk of comorbid Cog/PhysI increased at a rate of 7.37%, 95% CI [6.31%, 8.45%], per year of age. Specifically, WTC responders with compared to without PTSD had more than 3 times increased risk for co‐occurring Cog/PhysI, a*RR* = 3.295, 95% CI [2.444, 4.441]. Nonimpaired responders were the youngest, followed by responders with CogI alone and those with PhysI or comorbid Cog/PhysI. In this population, CogI was associated with all measures of PhysI: The risk of CogI increased with evidence of reduced hand‐grip strength, a*RR* = 1.82, 95% CI [1.58, 2.09], *p* < .001; chair‐rise speed, a*RR* = 1.31, 95% CI [1.14, 1.50], *p* < .001; and walking speed, a*RR* = 1.74, 95% CI [1.44, 2.09]; *p* < .001; as well as with balance impairment, a*RR* = 1.97, 95% CI [1.61, 2.41], *p* < .001.

**Table 1:** Characteristics of World Trade Center Responders Who Participated in Both Cognitive and Physical Aging Studies

	Normal			Cognitive impairment only			Physical impairment only			Cognitive and physical impairment		
	(*n* = 2,960)			(*n* = 375)			(*n* = 1,200)			(*n =* 280)		
Characteristic	*M*	*SD*	%	*M*	*SD*	%	*M*	*SD*	%	*M*	*SD*	%
Age (years)	51.68	7.53		53.88	7.62		55.02	8.12		58.00	9.01	
Maximal hand grip strength (lb)	84.83	25.80		76.36	21.65		64.32	29.51		57.54	25.85	
Walking speed (m/s)	1.14	0.16		1.12	0.18		0.98	0.20		0.93	0.20	
Chair‐rise speed (r/s)	0.56	0.14		0.55	0.13		0.39	0.12		0.40	0.12	
World Trade Center exposure severity	17.48	6.24		17.57	6.22		17.40	6.13		17.30	5.89	
Female			9.09			9.87			9.33			7.50
Posttraumatic stress disorder			9.93			13.33			18.25			30.00
Educational attainment												
High school or less			22.23			28.27			28.50			34.29
Some college			46.72			49.07			42.50			43.57
College degree			31.05			22.67			29.00			22.14
Race/ethnicity												
White			76.01			73.25			65.60			70.71
Black			3.11			4.33			6.40			7.14
Other			14.32			14.67			18.40			14.64
Hispanic			6.55			7.75			9.60			7.50
Any World Trade Center injury			13.48			17.17			14.67			17.86
World Trade Center head injury/illness			1.42			2.00			2.40			3.93
>5 Weeks on the pit/pile^a^			51.49			53.58			59.47			57.50
Hypertension			29.63			39.08			36.00			45.00
Heart disease			11.76			18.58			9.60			20.71
Diabetes			5.88			11.42			6.40			18.21

*Note*. ^a^Refers to the debris that resulted from the collapse of the World Trade Center towers.

To highlight the overall prevalence of reduced functioning on motor tasks that might be associated with comorbid Cog/PhysI severity, Table 2 presents the occurrence of impairments across motor tasks associated with cognitive functioning and aging. Declines in chair‐rise speed were the most common, followed by reductions in hand‐grip strength, reductions in walking speed, and balance impairment. Follow‐up analyses further revealed that among WTC responders with co‐occurring Cog/PhysI, PhysI was most common across indicators of chair‐rise speed and hand grip strength.

**Table 2:** Prevalence of subdomains of Physical Functional Limitations Among Responders with Comorbid Cognitive and Physical Limitations

	Responders with comorbid cognitive and physical impairments who have each limitation type		Responders with comorbid cognitive and physical impairments who only have the listed limitation	
Motor task	%	95% CI	%	95% CI
Walk limitations	28.21	[23.02, 33.88]	6.07	[3.58, 9.54]
Balance limitations	20.71	[16.12, 25.94]	5.36	[3.03, 8.68]
Chair‐rise limitations	61.07	[55.09, 66.82]	25.71	[20.70, 31.25]
Hand‐grip limitations	47.50	[41.53, 53.53]	23.21	[18.40, 28.61]

*Note*. *N* = 280.

As shown in Table 3, multivariable‐adjusted multinomial logistic regression determined that responders with PTSD had 2 times the risk of PhysI and an increased risk of comorbid Cog/PhysI compared to those without PTSD. In addition, PTSD was associated with an increased risk of CogI. However, these associations were less robust.

**Table 3:** Age‐ and Sex‐Adjusted Risk Ratios (aRRs) Examining the Associations Among Posttraumatic Stress Disorder Status, World Trade Center Exposure Factors, and PTSD Symptoms and the Risks of Cognitive and Physical Impairment

	Physical impairment only		Cognitive impairment only		Comorbid cognitive and physical impairment		
Model	a*RR* *^b^*	95% CI	a*RR* *^b^*	95% CI	a*RR* *^b^*	95% CI	∼*R* ^2b^
PTSD							
Unadjusted	2.025***	[1.675, 2.448]	1.391*	[1.009, 1.918]	3.888***	[2.931, 5.157]	.011
Demographically adjusted	1.891***	[1.558, 2.296]	1.270	[0.918, 1.756]	3.425***	[2.556, 4.588]	.043
Fully adjusted*	1.831***	[1.503, 2.230]	1.254	[0.903, 1.742]	3.295***	[2.444, 4.441]	.050
WTC exposure^a^							
WTC exposure severity	0.987*	[0.974, 0.999]	0.988	[0.968, 1.007]	0.973*	[0.950, 0.996]	
> 5 weeks on‐site	1.110	[0.953, 1.293]	1.425**	[1.118, 1.816]	1.365*	[1.028, 1.813]	
WTC injury	1.173	[0.960, 1.431]	1.052	[0.759, 1.458]	1.072	[0.746, 1.540]	
WTC head injury	0.901	[0.527, 1.543]	1.480	[0.689, 3.179]	1.415	[0.664, 3.014]	
PTSD symptoms							
Unadjusted							.021
Reexperiencing	1.350	[0.976, 1.868]	1.508	[0.892, 2.551]	4.366***	[2.625, 7.260]	
Avoidance	0.914	[0.724, 1.155]	0.861	[0.587, 1.261]	0.908	[0.615, 1.342]	
Emotional numbing	1.706**	[1.180, 2.465]	0.972	[0.527, 1.790]	1.235	[0.679, 2.246]	
Hyperarousal	1.376*	[1.033, 1.834]	1.332	[0.841, 2.111]	1.128	[0.688, 1.848]	
Demographically adjusted							.051
Reexperiencing	1.263	[0.908, 1.757]	1.375	[0.809, 2.338]	3.868***	[2.288, 6.538]	
Avoidance	0.976	[0.770, 1.237]	0.901	[0.612, 1.327]	1.081	[0.722, 1.618]	
Emotional numbing	1.534*	[1.055, 2.228]	0.919	[0.496, 1.701]	0.977	[0.526, 1.818]	
Hyperarousal	1.407*	[1.052, 1.884]	1.324	[0.832, 2.108]	1.168	[0.705, 1.937]	
Fully adjusted							.058
Reexperiencing	1.236	[0.886, 1.723]	1.347	[0.79, 2.295]	3.962***	[2.327, 6.745]	
Avoidance	1.007	0.793, 1.278]	0.897	[0.608, 1.325]	1.102	[0.734, 1.655]	
Emotional numbing	1.570*	[1.079, 2.285]	0.900	[0.485, 1.672]	0.971	[0.519, 1.817]	
Hyperarousal	1.322	[0.986,1.773]	1.353	[0.849, 2.158]	1.105	[0.663, 1.841]	

*Note*. WTC = World Trade Center. Unadjusted analyses do not account for any covariates; minimally adjusted analyses incorporated age, sex, education, and race/ethnicity; and fully adjusted models additionally account for WTC exposure severity, any WTC‐related injury, WTC‐related head injuries or illness, hypertension, heart problems, and diabetes; ^a^Exposure models report results from matching fully adjusted PTSD models. ^b^Covariable‐adjusted risk ratio. Adjusted relative risk ratios provide the likelihood of an event occurring in the exposure group in comparison to the likelihood occurring in the referent group. As a measure of effect size, an a*RR* value less than 1.00 indicates the risk is decreased, and an a*RR* value greater than 1.00 indicates an increased risk. ^c^Value can range from 0 to 1 and compares the likelihood for the intercept only model to the likelihood for the model with the predictors.

^*^ *p* < .05. ^**^ *p* < .01. ^***^ *p* < .001.

A second model in Table 3 (full estimates are provided in the Supplementary Materials) examining the unique contribution of PTSD symptom domains to PhysI, CogI, and comorbid Cog/PhysI revealed that the emotional numbing and reexperiencing symptom domains were independently associated with an increased risk of PhysI and comorbid Cog/PhysI, respectively. In unadjusted models, WTC responders with higher levels of emotional numbing symptoms had a 181% increase in the risk of PhysI compared to responders who endorsed PTSD across other domains. After adjusting for covariates, including age and sex, the risk of PhysI remained significant, although less robust. However, in fully adjusted models that accounted for sociodemographic factors and included the severity of WTC‐related exposures and medical comorbidities (i.e., hypertension, diabetes), emotional numbing symptoms were no longer a significant predictor of PhysI. Consistent with previous work, however, the analyses did identify an association between working more than 5 weeks on the pit/pile and an increased risk of CogI alone as well as with an increased risk of comorbid Cog/PhysI, although pulmonary exposure severity was protective. The fully adjusted models identified associations between an increased risk of (a) PhysI alone and age, Hispanic ethnicity, heart problems, and diabetes; (b) CogI alone and age, race/ethnicity, and lower educational attainment; and (c) comorbid Cog/PhysI and older age, Black race, diabetes, and lower educational attainment.

Across all models, the reexperiencing symptom domain remained a robust predictor for comorbid Cog/PhysI (see Table 3). Responders who endorsed higher levels of reexperiencing symptoms had a 4‐fold increase in the risk for comorbid Cog/PhysI in the unadjusted models. In subsequent models that accounted for sociodemographic factors and WTC‐related exposures as well as comorbid medical conditions, the increase in risk remained highly significant.

## Discussion

In the aftermath of the 9/11 terrorist attacks, responders worked tirelessly as part of the search, rescue, and recovery operations at the WTC site. Thousands of individuals inhaled an array of toxins (e.g., clouds of dust, burning fuel) and endured physical (e.g., digging through rubble, hauling debris) and repeated exposures to traumatic and stressful events (e.g., exposure to blood, human remains). These WTC responders are now approaching midlife. Converging evidence indicates that PTSD remains among the top five psychiatric conditions reported by WTC‐exposed individuals. Further, responders with chronic PTSD have an increased risk for impairments across both cognitive and physical domains of functioning, both of which are commonly associated with older age and neurodegenerative conditions (Clouston, Diminich, et al., 2019; Clouston, Guralnik, et al., 2017; Mukherjee et al., 2019).

Increasingly, PTSD, a psychiatric condition with marked heterogeneity in symptom presentation, has been linked with CogI (Schuitevoerder et al., 2013), often a prodrome for dementia, and PhysI (Hedden & Gabrieli, 2004). Thus, given the considerable evidence indicating that chronic stress may influence aging processes (Miller & Sadeh, 2014), we examined the extent to which PTSD was associated with CogI, PhysI, and co‐occurring Cog/PhysI among WTC responders. The results of the present study indicate that although the mean age of the responders included in the present analyses was 54 years, WTC responders with PTSD have an increased risk of PhysI and face an increased risk for co‐occurring Cog/PhysI more commonly seen in older adults. Notably, the strength of these associations remained after adjusting for a number of covariates, including educational attainment and cardiovascular disease, both of which are risk factors for neurodegenerative disorders.

The present findings further reveal differences across motor tasks associated with cognitive functioning and aging, which may be important comorbidity with CogI in this cohort. A reduction in hand‐grip strength was the most common comorbid condition with CogI, followed closely by reduced chair‐rise speed. In addition, the results revealed that of responders with CogI, nearly half also had PhysI. Previous work has found that reductions in grip strength are a rigorous biomarker for aging (Sanderson & Scherbov, 2014) that is related to CogI (Duggan et al., 2018, 2019) and is highly associated with PTSD (Mukherjee et al., 2019) as well as an increased risk of mild cognitive impairment, a transient stage between normative aging and dementia (Boyle et al., 2010). Walking speed is a good predictor of the risk of mortality (Studenski et al., 2011) and is associated with neuroinflammatory diseases. Indeed, PhysI is a common hallmark of neurodegenerative diseases including, for example, amyotrophic lateral sclerosis and Parkinson's‐type dementias (Boyle et al., 2009, 2010).

A hallmark of PTSD is its complexity in presentation, with symptoms fluctuating over time. Our findings are largely consistent with existing research conducted in veteran populations that has demonstrated unique associations with emotional numbing symptoms and epigenetic aging (Wolf et al., 2019) as well as self‐reported impairments across social and occupational functioning (Shea et al., 2010). Although we did not find associations between symptoms of hyperarousal or avoidance with PhysI, CogI, or comorbid Cog/PhysI, which previous work has indicated are predictive of higher levels of systemic inflammation (Passos et al., 2015) and heightened autonomic reactivity (Yehuda et al., 2015), we propose that these differences are likely attributed to the range of WTC‐exposures experienced versus combat exposure and individual differences within the WTC‐cohort relative to veterans and military personnel. Collectively, these findings underscore the importance of chronic PTSD as a significant risk factor for earlier onset PhysI and comorbid Cog/PhysI, more commonly seen in older adults, among WTC rescue and recovery workers and further highlight the unique associations between specific PTSD symptoms and long‐term physical and cognitive functioning.

Neurobiological models of PTSD highlight the role of prolonged stress, with dysregulated physiological processes across biological systems associated with inflammation and structural and functional brain changes (Lohr et al., 2015; Picard & McEwen, 2018). Previous research has linked PTSD to hippocampal and amygdala volume reduction (Gilbertson et al., 2002; O'Doherty et al., 2015); white matter lesions (Pitman et al., 2001); white matter integrity, diffusivity, and anisotropy (Daniels et al., 2013; Davenport et al., 2015); glucose uptake in the amygdala (Buchsbaum et al., 2015; Zhu et al., 2016); and neuroinflammation and microglial activation (Seibyl, 2012). Thus, researchers increasingly posit that PTSD is a multisystemic disorder whereby reactivation of trauma memories results in elevated levels of inflammation, exacerbating symptoms of aging‐related diseases in the body, with prolonged stress accelerating cellular aging and apoptosis and resulting in more rapid aging (Greenberg et al., 2014) and premature mortality (Lohr et al., 2015).

Taken together, these analyses support the view that chronic PTSD may be related to an increased risk of comorbid Cog/PhysI, for which deficits across motor tasks, namely reductions in hand‐grip strength and chair‐rise speed may act as prodromal indicators. Because cognitively impairing conditions usually arise after substantial losses in brain matter are evident, these results may suggest that investigators interested in understanding the risk of PTSD with co‐morbid Cog/PhysI may need to better understand the extent to which neurodegeneration is evident in prodromal PhysI. Future work is needed to improve the characterization of the prodromal condition.

The present results should be considered within the context of several limitations. It may be difficult to generalize the findings, as responders were predominantly employed in law enforcement, male, self‐identified as White, and comprised a population of rescue and recovery workers exposed to the historical events of the 9/11 terrorist attacks. Therefore WTC‐exposed individuals are not representative of the general population and may not be similar to other first‐responder and nontraditional workers and volunteer populations. Nonetheless, this sample did not differ regarding age, exposure levels, and levels of posttraumatic stress in relation to other WTC cohorts (Dasaro et al., 2017). In addition, the WTC responders in the present sample may be subject to selection bias, as they are made up of well‐educated, relatively healthy individuals who voluntarily enrolled in the WTC‐HP for monitoring after working at the WTC. Furthermore, many responders, such as police officers and firefighters, likely participated in disaster training as required by various employers and agencies prior to 9/11, which potentially enhanced resilience against the multitude of psychological stressors while working on‐site at the WTC.

As noted in previous work (Clouston, Diminich, et al., 2019; Clouston, Guralnik, et al., 2017; Clouston, Pietrzak, et al., 2017), inhalation of fine airborne particles and dust exposure while on‐site may indeed have contributed to an increased risk of CogI. However, as previously theorized, it is likely this risk is most commonly due to an increased risk of inhaled particulate matter including—notably, particulate matter less than 2.5 mm in size (Haghani et al., 2020). Responders were exposed to a large amount of inhaled particulate matter that was aerosolized for months following the WTC disaster (Lioy & Georgopoulos, 2006). The role of WTC‐related environmental exposures and subsequent exposures to physical stressors in relation to PhysI, CogI, or comorbid Cog/PhysI was not explored in the present study. Moreover, WTC‐related environmental exposures have been associated with a number of age‐related diseases, including poor pulmonary functioning (Banauch et al., 2005), increased incidence of cancer (Li et al., 2012), and aerodigestive disease (Wisnivesky et al., 2011). In the present study, we replicated earlier results showing that the length of time spent on the pit/pile at the WTC site was associated with CogI, but we additionally showed that the profile was somewhat different from PTSD‐related CogI in that WTC exposures were associated with both CogI alone and comorbid Cog/PhysI. This was the first study of which we are aware to also report that overall pulmonary exposures were protective. This may not be surprising: The most severe pulmonary exposures occurred in the day of the attacks, when airborne particulate matter was coarsest. During this time, many responders were so severely exposed that they did not return to the site. However, our findings suggest that the increased risk for PhysI and comorbid Cog/PhysI remained highly significant in models that were fully adjusted for the severity of WTC exposures; exposure information was collected retrospectively and may have been subject to recall bias.

The present study did not assess the extent to which physical and/or psychological stressors in subsequent years may have been associated with PhysI, CogI, or comorbid Cog/PhysI among responders. We also did not account for the age CogI or PhysI onset, as the measures were not part of the broader monitoring program. Although longitudinal work has identified an increased incidence of CogI in WTC responders with PTSD (Clouston, Diminich, et al., 2019), to date, little is known about changes in PhysI. The analyses were not adjusted for the presence and time of onset of other psychiatric and medical conditions in individuals with CogI, PhysI, neither, or both (Cog/PhysI). Future analyses will examine the contributions of specific medical conditions prevalent in WTC cohorts.

In the present study, we examined how PTSD and its symptom domains were associated with an increased risk of CogI, PhysI, and/or comorbid Cog/PhysI in responders to the 9/11 terrorist attacks on the WTC in New York City. When individuals were differentiated into groups based on the presence of PhysI, CogI, and comorbid Cog/PhysI, significant associations emerged, with PTSD as a risk factor for both PhysI and comorbid Cog/PhysI. The analyses revealed that PTSD remained a strong predictor of Cog/PhysI, with focal effects in the reexperiencing symptom domain. It is important to note that robust associations between PTSD and both PhysI and comorbid Cog/PhysI remained after controlling for vascular risk factors and disease burden that can account for an increased risk of neurodegenerative disease. The results may suggest that WTC responders with chronic PTSD have an increased risk for more than age‐expected deficits across cognitive and functional domains, which may be indicative of a multisystemic disorder characterized by the emergence of Cog/PhysI at midlife. More research is warranted to understand the links between chronic PTSD and the presence of aging‐related conditions.

## Open Practices Statement

The study reported in this article was not formally preregistered. Due to the sensitivity of medical diagnoses and the use of dates in this study, data and materials have not been made available on a permanent third‐party archive. Data and material requests should be sent via email to the corresponding author at Sean.C louston@stonybrookmedicine.edu

## Supporting information

Supporting Material
