# Supplementary material for: Chronic Posttraumatic Stress Disorder and Comorbid Cognitive and Physical Impairments in World Trade Center Responders
Source: J Trauma Stress. 2020 Nov 21;34(3):616–27. doi: 10.1002/jts.22631 (PMC8137717; doi:10.1002/jts.22631)
Supplement: Supplementary file 1 — Supporting Material [file JTS-34-616-s001.docx]

**Supplemental Appendix A**

**Table A.1.** Complete results from multivariable-adjusted multinomial logistic regression models reporting adjusted risk ratios and 95% confidence intervals for all covariates included in model

|  |  | Unadjusted (PI) | | | | |  | | | Demographically Adjusted (PI) | | | | |  | | | Fully Adjusted (PI) | | | | |
| --- | --- | --- | --- | --- | --- | --- | --- | --- | --- | --- | --- | --- | --- | --- | --- | --- | --- | --- | --- | --- | --- | --- |
| Physical Impairment |  | aRR | | 95% C.I. | |  | | | aRR | | | 95% C.I. | |  | | | aRR | | | 95% C.I. | |  |
| Posttraumatic Stress Disorder |  | 2.025 | | 1.675-2.448 | |  | | | 1.891 | | | 1.558-2.296 | |  | | | 1.831 | | | 1.503-2.23 | |  |
| Age |  |  | |  | |  | | | 1.054 | | | 1.045-1.064 | |  | | | 1.047 | | | 1.038-1.057 | |  |
| Female |  |  | |  | |  | | | 1.097 | | | 0.861-1.397 | |  | | | 1.112 | | | 0.872-1.419 | |  |
| Race/Ethnicity |  |  | |  | |  | | |  | | |  | |  | | |  | | |  | |  |
| White |  |  | |  | |  | | | 1.000 | | |  | |  | | | 1.000 | | |  | |  |
| Black |  |  | |  | |  | | | 1.445 | | | 1.007-2.073 | |  | | | 1.397 | | | 0.971-2.01 | |  |
| Other |  |  | |  | |  | | | 1.117 | | | 0.917-1.361 | |  | | | 1.156 | | | 0.944-1.415 | |  |
| Hispanic |  |  | |  | |  | | | 1.315 | | | 1.008-1.715 | |  | | | 1.318 | | | 1.009-1.721 | |  |
| Hypertension |  |  | |  | |  | | |  | | |  | |  | | | 1.152 | | | 0.99-1.341 | |  |
| Heart Problems |  |  | |  | |  | | |  | | |  | |  | | | 1.318 | | | 1.085-1.601 | |  |
| Diabetes |  |  | |  | |  | | |  | | |  | |  | | | 1.538 | | | 1.201-1.969 | |  |
| WTC Exposure Severity |  |  | |  | |  | | |  | | |  | |  | | | 0.988 | | | 0.976-1 | |  |
| More than 5 weeks on-site |  |  | |  | |  | | |  | | |  | |  | | | 1.112 | | | 0.955-1.294 | |  |
| WTC Injury |  |  | |  | |  | | |  | | |  | |  | | | 1.227 | | | 1.006-1.495 | |  |
| WTC Head Injury |  |  | |  | |  | | |  | | |  | |  | | | 0.992 | | | 0.583-1.689 | |  |
| Educational Attainment |  |  | |  | |  | | |  | | |  | |  | | |  | | |  | |  |
| High School or less |  |  | |  | |  | | | 1.000 | | |  | |  | | | 1.000 | | |  | |  |
| Some College |  |  | |  | |  | | | 0.791 | | | 0.668-0.938 | |  | | | 0.780 | | | 0.658-0.925 | |  |
| University Degree or more |  |  | |  | |  | | | 0.827 | | | 0.687-0.995 | |  | | | 0.823 | | | 0.683-0.992 | |  |
|  |  |  | |  | |  | | |  | | |  | |  | | |  | | |  | |  |
| Cognitive Impairment |  |  | |  | |  | | |  | | |  | |  | | |  | | |  | |  |
| Posttraumatic Stress Disorder |  | 1.391 | | 1.009-1.918 | |  | | | 1.270 | | | 0.918-1.756 | |  | | | 1.254 | | | 0.903-1.742 | |  |
| Age |  |  | |  | |  | | | 1.039 | | | 1.024-1.053 | |  | | | 1.039 | | | 1.024-1.054 | |  |
| Female |  |  | |  | |  | | | 1.105 | | | 0.76-1.608 | |  | | | 1.130 | | | 0.775-1.648 | |  |
| Race/Ethnicity |  |  | |  | |  | | |  | | |  | |  | | |  | | |  | |  |
| White |  |  | |  | |  | | |  | | |  | |  | | |  | | |  | |  |
| Black |  |  | |  | |  | | | 2.402 | | | 1.486-3.88 | |  | | | 2.266 | | | 1.397-3.674 | |  |
| Other |  |  | |  | |  | | | 1.537 | | | 1.152-2.051 | |  | | | 1.491 | | | 1.108-2.007 | |  |
| Hispanic |  |  | |  | |  | | | 1.771 | | | 1.208-2.597 | |  | | | 1.751 | | | 1.193-2.569 | |  |
| Hypertension |  |  | |  | |  | | |  | | |  | |  | | | 1.145 | | | 0.902-1.453 | |  |
| Heart Problems |  |  | |  | |  | | |  | | |  | |  | | | 0.690 | | | 0.476-1.001 | |  |
| Diabetes |  |  | |  | |  | | |  | | |  | |  | | | 0.891 | | | 0.567-1.401 | |  |
| WTC Exposure Severity |  |  | |  | |  | | |  | | |  | |  | | | 0.988 | | | 0.969-1.008 | |  |
| More than 5 weeks on-site |  |  | |  | |  | | |  | | |  | |  | | | 1.423 | | | 1.117-1.813 | |  |
| WTC Injury |  |  | |  | |  | | |  | | |  | |  | | | 1.079 | | | 0.779-1.494 | |  |
| WTC Head Injury |  |  | |  | |  | | |  | | |  | |  | | | 1.549 | | | 0.724-3.313 | |  |
| Educational Attainment |  |  | |  | |  | | |  | | |  | |  | | |  | | |  | |  |
| High School or less |  |  | |  | |  | | | 1.000 | | |  | |  | | | 1.000 | | |  | |  |
| Some College |  |  | |  | |  | | | 0.887 | | | 0.684-1.149 | |  | | | 0.886 | | | 0.683-1.149 | |  |
| University Degree or more |  |  | |  | |  | | | 0.621 | | | 0.457-0.844 | |  | | | 0.629 | | | 0.462-0.856 | |  |
|  |  |  | |  | |  | | |  | | |  | |  | | |  | | |  | |  |
| Cognitive and Physical Impairment | | |  | |  | | |  | | |  | |  | | |  | | |  | |  |  |
| Posttraumatic Stress Disorder |  | 3.888 | | 2.931-5.157 | |  | | | 3.425 | | | 2.556-4.588 | |  | | | 3.295 | | | 2.444-4.441 | |  |
| Age |  |  | |  | |  | | | 1.098 | | | 1.082-1.115 | |  | | | 1.089 | | | 1.071-1.106 | |  |
| Female |  |  | |  | |  | | | 0.889 | | | 0.546-1.447 | |  | | | 0.931 | | | 0.57-1.521 | |  |
| Race/Ethnicity |  |  | |  | |  | | |  | | |  | |  | | |  | | |  | |  |
| White |  |  | |  | |  | | |  | | |  | |  | | |  | | |  | |  |
| Black |  |  | |  | |  | | | 2.607 | | | 1.521-4.468 | |  | | | 2.422 | | | 1.404-4.177 | |  |
| Other |  |  | |  | |  | | | 1.158 | | | 0.804-1.666 | |  | | | 1.215 | | | 0.836-1.767 | |  |
| Hispanic |  |  | |  | |  | | | 1.345 | | | 0.823-2.197 | |  | | | 1.371 | | | 0.838-2.244 | |  |
| Hypertension |  |  | |  | |  | | |  | | |  | |  | | | 1.140 | | | 0.868-1.498 | |  |
| Heart Problems |  |  | |  | |  | | |  | | |  | |  | | | 1.233 | | | 0.882-1.724 | |  |
| Diabetes |  |  | |  | |  | | |  | | |  | |  | | | 2.187 | | | 1.518-3.152 | |  |
| WTC Exposure Severity |  |  | |  | |  | | |  | | |  | |  | | | 0.976 | | | 0.954-0.999 | |  |
| More than 5 weeks on-site |  |  | |  | |  | | |  | | |  | |  | | | 1.389 | | | 1.047-1.842 | |  |
| WTC Injury |  |  | |  | |  | | |  | | |  | |  | | | 1.137 | | | 0.794-1.627 | |  |
| WTC Head Injury |  |  | |  | |  | | |  | | |  | |  | | | 1.738 | | | 0.831-3.637 | |  |
| Educational Attainment |  |  | |  | |  | | |  | | |  | |  | | |  | | |  | |  |
| High School or less |  |  | |  | |  | | | 1.000 | | |  | |  | | | 1.000 | | |  | |  |
| Some College |  |  | |  | |  | | | 0.734 | | | 0.547-0.983 | |  | | | 0.723 | | | 0.538-0.971 | |  |
| University Degree or more |  |  | |  | |  | | | 0.586 | | | 0.414-0.828 | |  | | | 0.595 | | | 0.42-0.844 | |  |
